# Disentangling Hippocampal Shape Anomalies in Epilepsy

**DOI:** 10.3389/fneur.2013.00131

**Published:** 2013-09-11

**Authors:** Hosung Kim, Tommaso Mansi, Neda Bernasconi

**Affiliations:** ^1^Neuroimaging of Epilepsy Laboratory, McConnell Brain Imaging Center, Montreal Neurological Institute and Hospital, McGill University, Montreal, QC, Canada; ^2^Imaging and Computer Vision, Corporate Technology, Siemens Corporation, Princeton, NJ, USA

**Keywords:** temporal lobe epilepsy, malformations of cortical development, hippocampus, image analysis, shape analysis

## Abstract

Drug-resistant temporal lobe epilepsy (TLE) and epileptic syndromes related to malformations of cortical development (MCD) are associated with complex hippocampal morphology. The contribution of volume and position to the overall hippocampal shape in these conditions has not been studied. We propose a surface-based framework to localize volume changes through measurement of Jacobian determinants, and quantify fine-scale position and curvature through a medial axis model. We applied our methodology to T1-weighted 3D volumetric MRI of 88 patients with TLE and 78 patients with MCD, including focal cortical dysplasia (FCD, *n* = 29), heterotopia (HET, *n* = 40), and polymicrogyria (PMG, *n* = 19). Patients were compared to 46 age- and sex-matched healthy controls. Surface-based analysis of volume in TLE revealed severe ipsilateral atrophy mainly along the rostro-caudal extent of the hippocampal CA1 subfield. In MCD, patterns of volume changes included bilateral CA1 atrophy in HET and FCD, and left dentate hypertrophy in all three groups. The analysis of curvature revealed medial bending of the posterior hippocampus in TLE, whereas in MCD there was a supero-medial shift of the hippocampal body. Albeit hippocampal shape anomalies in TLE and MCD result from a combination of volume and positional changes, their nature and distribution suggest different pathogenic mechanisms.

## Introduction

Growing evidence suggests that abnormalities of brain development are etiological factors contributing to epilepsy. In particular, syndromes in which pathogenic mechanisms involve the temporal lobe may be associated with abnormal hippocampal morphology, identified as atypical shape and positioning. These macroscopic markers of brain maldevelopment, referred to as malrotation, have been reported in high proportions of patients with temporal lobe epilepsy (TLE) and those with malformation of cortical development (MCD) (1–7). Such anomalies may be found in about 10% of healthy controls, and are believed to represent the end of phenotypic spectrum of a normal hippocampal formation (3, 8). In our previous work, we created quantitative models of hippocampal malrotation based on their MRI characteristics (7, 8). These empirically defined models were designed to assess at a global scale hippocampal shifting and rotation with respect to its long axis, and thus only partially captured the complexity of hippocampal morphology.

In this work, we aimed at assessing independently the contribution of volume and position to the overall hippocampal shape in TLE and MCD. To this purpose, we applied our previously developed and validated methods (9, 10), based on spherical harmonic shape descriptors. Our data-driven approach localized variations of volume at a sub-millimetric level through the analysis of Jacobian determinants, and quantified fine-scale local position and curvature through a medial axis model.

## Materials and Methods

### Subjects

From our database of patients referred for the investigation of drug-resistant epilepsy, we selected patients with MCD (*n* = 88; 39 males; mean age: 31 ± 9, range: 18–58) and those with TLE (*n* = 78; 34 males; mean age: 36 ± 10, range: 17–57) who had been examined with the same MRI scanner and in whom hippocampal volumetry was performed. Patients in whom the cortical developmental malformation invaded the hippocampus were excluded. No TLE patient had a mass lesion (tumor, vascular malformation) or traumatic brain injury. The MCD population included patients with focal cortical dysplasia (*n* = 29; 11 males; mean age: 30 ± 9), heterotopia (*n* = 40; 19 males; mean age: 31 ± 10), and polymicrogyria (*n* = 19; 9 males; mean age: 32 ± 8). Focal cortical dysplasia was located outside the temporal lobe in all patients. The heterotopia group included patients with double cortex (*n* = 11), periventricular nodular heterotopia (*n* = 26; bilateral in 13, unilateral in 13), and unilateral subcortical heterotopia (*n* = 3). Lesions of polymicrogyria were bilateral in 16/19 patients (perisylvian in 12, other combinations in 4), and unilateral perisylvian in 3/19.

Demographic and clinical data were obtained through interviews with the patients and their relatives. The diagnosis and localization of the seizure focus was based on a standard clinical evaluation including detailed history, seizure semiology, neurological examination, video-EEG recordings, and MRI evaluation in all patients.

In TLE, the seizure focus was left-sided in 34 patients and right-sided in 44. Sixty-seven patients underwent surgery. Mean ± SD follow-up time was 3.6 ± 3.2 years. We determined post-surgical seizure outcome according to Engel’s modified classification (11) 51 (76%) patients had Class I outcome, 5 (8%) Class II, 7 (10%) Class III, and 4 (6%) Class IV. In MCD patients, the seizure focus was frontal in 15 patients, parietal in 7, temporal in 21, and multilobar or generalized in 37. Five patients presented only with interictal slow activity and three patients had a normal EEG.

The control group consisted of 46 age- and sex-matched (23 males; mean age: 32 ± 12 years, range: 20–56) healthy individuals. The Ethics Committee of the Montreal Neurological Institute and Hospital approved the study and written informed consent was obtained from all participants.

### MRI acquisition and pre-processing

Images were acquired on a 1.5T Gyroscan (Philips Medical Systems, Eindhoven, Netherlands) using a 3D T1-fast field echo sequence (TR = 18 ms; TE = 10 ms; NEX = 1; flip angle = 30°; matrix size = 256 × 256; FOV = 256 mm; slice thickness = 1 mm), providing an isotropic voxel volume of 1 mm^3^. Prior to processing, images underwent automated correction for intensity non-uniformity and intensity standardization (12).

The hippocampus was segmented manually according to our previously published protocol (13). Prior to segmentation, MR images were registered into the MNI ICBM-152 template (14) using nine parameter linear transformation (15). Hippocampal volumes were normalized through a *z*-transformation relative to the corresponding distribution of healthy controls.

### Surface-based analysis of volume

Hippocampal labels were converted to surface meshes and parameterized using spherical harmonics with point distribution model (SPHARM-PDM) (16). The SPHARM-PDM surfaces of each individual were rigidly aligned to a hippocampal template (constructed from the mean surface of controls and patients) using their centroid and the longitudinal axis of the first order ellipsoid (17). To correct for differences in overall head size, each surface was inversely scaled with respect to intra-cranial volume (16). We calculated displacement vectors between each subject’s SPHARM-PDM surface and the template across 1,002 vertices (16). The signed surface normal components of these vectors quantify inward/outward deformations. To compute vertex-wise volume changes, we applied the heat equation to interpolate the vertex-wise displacement vectors within the volume enclosed by the SPHARM-PDM surface (9). The Jacobian determinant *J* of the resulting dense vector field was projected back onto the surface to quantify growth (*J*−1 > 0) or shrinkage (*J*−1 < 0) along the surface normal direction.

### Construction of the medial axis

**Figure 1:**
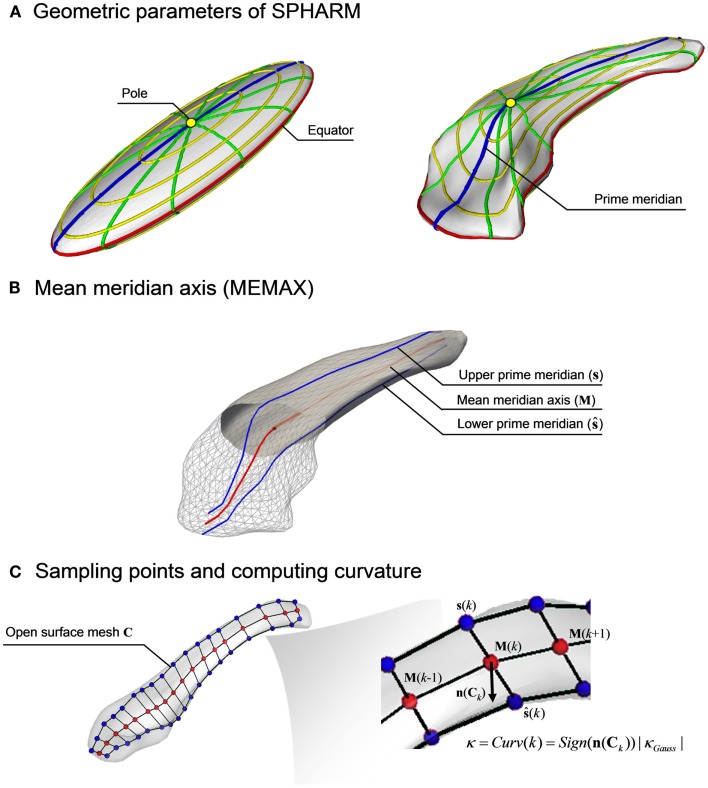
**Computation of the mean meridian axis**. **(A)** The SPHARM function defined by two spherical harmonic coefficients, the latitudes (yellow) and the longitudes (green), are mapped on the first degree ellipsoid surface. The prime meridian (blue) defines the longitude when ϕ = 0 or π. After computing spherical basis functions for the hippocampus, the coefficients are repositioned while preserving shape. **(B)** The geometric mean of the upper (**s**) and lower (**ŝ**) prime meridians generates the mean meridian axis (MEMAX, **M**). **(C)** Using an equidistant subdivision of the latitudes, *K* points are sampled on **M**. After creating an open surface mesh **(C)**, whose boundaries are defined by the prime meridians **s** and **ŝ**, the signed Gaussian curvature (κ_Gauss_) is computed at each sample point **M**(*k*) through the surface normal vector ***n***(**C**_k_).

According to the SPHARM parameterization (16), the coordinates of a given point on the surface is defined by parameters representing its latitude and longitude (Figure 1A). Varying the latitude from 0 to 180°at the location of the 0°longitude describes a set of points that constitute the prime meridian on a hemisphere. In the same manner, their counterpart points on the opposite hemisphere are obtained by varying the latitude at the 180°longitude. Averaging these two sets of points in a pair-wise fashion yields a mean meridian axis, henceforth called MEMAX, representing a “skeleton” that inherits the point correspondence of the SPHARM-PDM (Figure 1B) (10). For finer analyses, we further resampled points on the MEMAX (Figure 1C) using equiangular subdivisions of the latitude.

To measure shifting, we conducted a group-wise comparison for each sample point position in the 3D space. To measure bending, we computed a signed curvature to distinguish convexity from concavity. Specifically, we first created an open surface mesh whose boundaries were defined by the prime meridian and that passes through the MEMAX. We then calculated the signed Gaussian curvature on that surface (the sign is given by the orientation of the normal vectors), and used the values at the points on the MEMAX.

### Statistical analysis

We compared surface-based point-wise volume and Gaussian curvatures between patient groups and controls using vertex-wise Student’s *t*-tests, and position vectors using point-wise Hotelling *T*^2^-test. In TLE, patients were assessed relative to the epileptogenic hemisphere (i.e., ipsilateral and contralateral to the seizure focus) by normalizing vertex-wise volume, curvature, and position vector using a *z*-transformation with respect to the distribution of the corresponding hemisphere of controls. Analyses were corrected for multiple comparisons using the false discovery rate (FDR) correction at *q* < 0.05 (18).

To facilitate the anatomical localization of results, we outlined hippocampal subfields on the surface template according to a histological parcellation atlas (19).

## Results

### Global volumetry

Based on 2 SD beyond mean absolute volume and inter-hemispheric asymmetry of healthy controls, all TLE patients showed hippocampal atrophy ipsilateral to the seizure focus.

In patients with MCD, volumetric anomalies were present in 51% (45/88) of patients and included both atrophy in 73% (33/45) and hypertrophy in 27% (12/45). Specifically, in focal cortical dysplasia, 34% (10/29) of patients had hippocampal atrophy (ipsilateral to the seizure focus in 80%) and 10% (3/29) had hippocampal hypertrophy (ipsilateral in 1, bilateral in 1, and contralateral in 1). In heterotopia, 43% (17/40) of patients had atrophy (unilateral in 11/17, bilateral in 6/17) and 10% (4/40) had hypertrophy (unilateral in 2, bilateral in 2). In polymicrogyria 32% (6/19) of patients had atrophy (unilateral in 4/6, bilateral in 2/6) and 26% (5/19) had hypertrophy (unilateral in 4, bilateral in 1).

### Surface-based analysis

**Figure 2:**
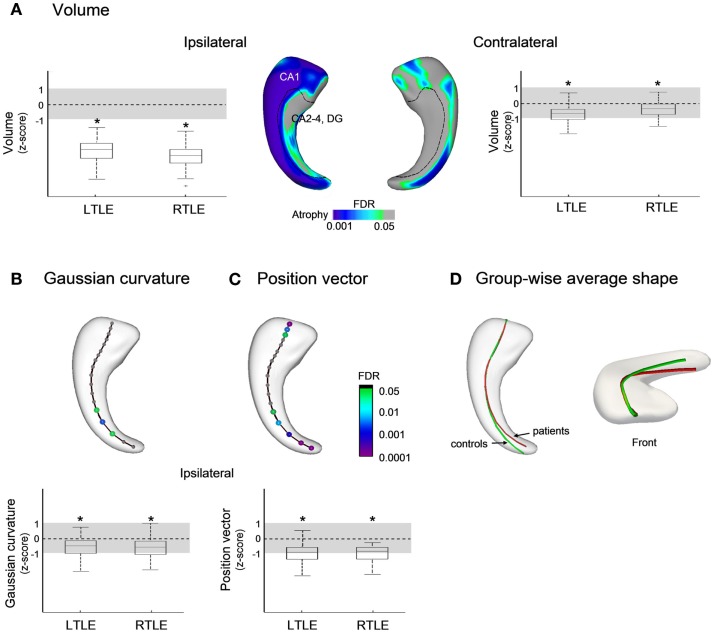
**Group comparisons between patients with temporal lobe epilepsy (TLE) and healthy controls for: (A) Volume; (B) Gaussian curvature; and (C) position vectors; in (D), the group-wise mean meridian axis clearly shows the infero-medial shifting of the hippocampus due to increased curvature in its caudal segment**. Box-and-whisker plots present group differences between each patient group standardized relative to healthy controls; the solid line indicates the mean and the dotted lines represent ±1 SD from the mean. Color-scales refer to FDR-corrected *p*-values, and L (left) and R (right) to seizure focus lateralization.

Compared to controls, surface-based analysis in TLE localized severe ipsilateral atrophy mainly along the rostro-caudal extent of hippocampal CA1 subfield, with similar effects in patients with left and right epileptic foci (Figure 2A, FDR < 0.0005). Contralateral atrophy was less severe and limited to small portions of CA1 (FDR < 0.05). We found a significant correlation between disease duration and areas of atrophy ipsilateral to the seizure focus (*r* = −0.4, FDR < 0.05).

Analysis of the curvature revealed bending of the ipsilateral hippocampal tail toward the mid-sagittal plane (FDR < 0.05 Figure 2B), and the position vectors showed a shift from the supero-lateral to infero-medial direction with similar effects in both TLE groups (FDR < 0.05; Figures 2C,D). No position anomalies were found contralateral to the seizure focus.

**Figure 3:**
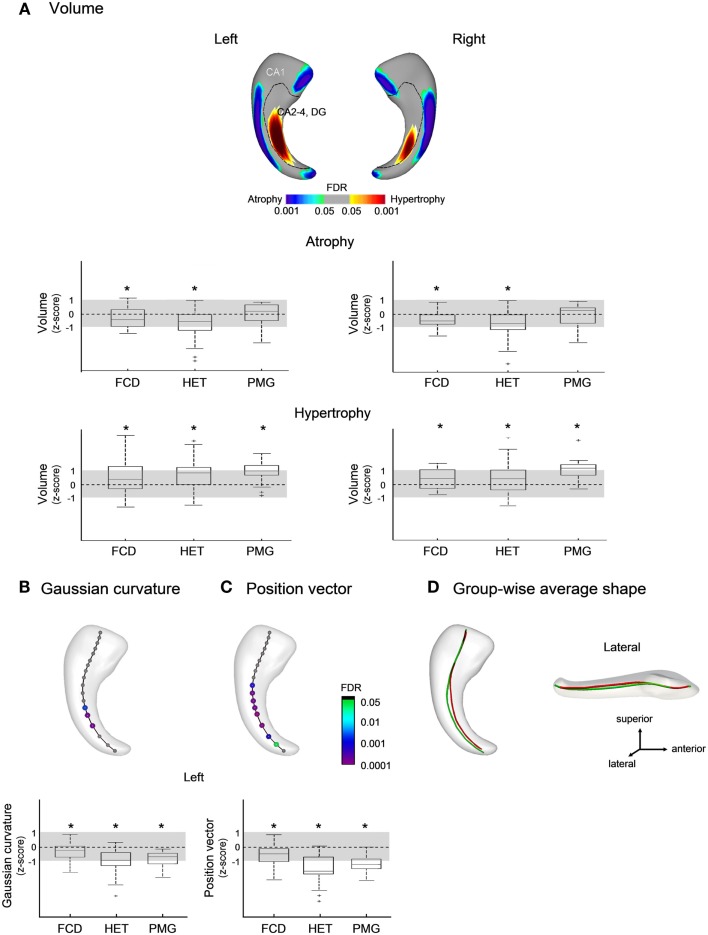
**Group comparisons between patients with malformations of cortical development (MCD) and healthy controls for: (A) volume; (B) Gaussian curvature; and (C) position vectors; in (D), the group-wise mean meridian axis shows a flattening (i.e., decrease in curvature) of the body along with a shift from the lateral to medial direction with respect to controls**. Box-and-whisker plots present group differences between each patient group standardized relative to healthy controls; the solid line indicates the mean and the dotted lines represent ± 1 SD from the mean. Color-scales refer to FDR-corrected *p*-values; FCD, focal cortical dysplasia; HET, heterotopia; PMG, polymicrogyria.

In patients with MCD (Figure 3A), we found bilateral symmetric CA1 atrophy in FCD (FDR < 0.05) and HET (FDR < 0.005). Overall, effects of atrophy were smaller in MCD than in TLE (FDR < 0.005). In addition, we found bilateral increased volume corresponding to CA4-DG (FDR < 0.05) in all three MCD groups. There was no correlation between areas of atrophy and disease duration.

The analysis of the medial axis detected anomalies in the left hippocampus only, with flattening (i.e., decrease in curvature) of the body (FDR < 0.001; Figure 3B) along with a shift from the lateral to medial direction compared to controls (FDR < 0.05; Figures 3C,D); the magnitude of these changes were more marked in HET and PMG than in FCD (FDR < 0.05).

## Discussion

Our study showed that morphological anomalies of the hippocampus in TLE and MCD result from a combination of volume and positional changes. Nevertheless, their nature and distribution differ between the two cohorts.

### Methodological considerations

Previous surface-based methods attempting to localize hippocampal structural changes have employed thickness-based measurements computed from medial surface or axis models (20–22). These approaches produce symmetrical measurements on facing surfaces and tend to mirror findings to the counterpart regions, challenging biologically plausible interpretations. Indeed, the subfields are not symmetrically distributed with respect to the geometric center of the hippocampus. Moreover, histopathological studies have shown that the magnitude and distribution of neuronal loss in TLE vary both within and across hippocampal subfields (23). Alternatively, by computing vertex-wise displacement vectors between a given object and a template, the SPHARM-PDM approach enables the localization of asymmetrical shape changes (22). The signed surface normal components of these vectors, signifying inward or outward deformation, can be interpreted as volume increase or decrease. A limitation, however, resides in the fact that this metric may also capture co-occurring positional differences, as we have previously shown (8). To obtain an actual metric of growth or shrinkage, we calculated the Jacobian determinants of the vector fields derived from SPHARM-PDM. We could thus detect asymmetrically distributed volume anomalies independent of positional variations, facilitating our interpretation of disease-related morphological anomalies.

A previous work has shown that registration procedure used in SPHARM-PDM may influence spatial metrics such as displacement and local positioning, particularly when dealing with shape variants (24). Notably, the shape descriptors we implemented, including Jacobian determinants to assess local volume and signed curvature to quantify bending, are intrinsically independent of the alignment process, as shown by our previous validation studies (8, 10).

### Biological consideration

In TLE, our analysis localized marked atrophy mainly in the ipsilateral CA1 together with a mild involvement of the other subfields, a finding in agreement with histopathological data (23). We also observed small areas of subtle contralateral atrophy confined to CA1. In MCD, volumetry of the hippocampus has been so far limited to a single study (25), in which proportions of patients displaying atrophy across various subgroups were similar to ours. Here, we refined the characterization of morphological changes by quantifying subregional anomalies and localized atrophy to the CA1 subfield, with a highly similar hemispheric distribution, although the overall extent and effects were smaller than in TLE. The co-existence of hippocampal atrophy and cortical developmental disorders, mainly heterotopias, has been defined as dual pathology (26, 27). The exact nature of this association is not clearly understood, particularly in patients without electroclinical features pointing to the involvement of the mesial temporal lobe. Nevertheless, electrophysiological studies targeting both the MCD lesion and the hippocampus suggest that the latter actively participates to the epileptogenic network and may not be an innocent bystander (28–31). Indeed, when the hippocampus is surgically resected, histopathology shows evidence for neuronal loss in CA1 (32). There is also indication for hippocampal hypoplasia as shown in postmortem analysis of human fetuses with doublecortin deficiency (33). In addition, in animal models of focal cortical malformations using freeze lesions acute changes in CA1 inhibitory network (34) have been followed by atrophy in the chronic phase (35). Nevertheless, the fact that the hippocampus is not the primary epileptogenic tissue in MCD may explain the lesser extent and degree of atrophy in these patients as compared to those with TLE. Further support comes from the lack of progression of atrophy in our MCD patients as opposed to TLE.

A striking difference between our two epilepsy cohorts consisted in the presence of bilateral hippocampal hypertrophy, which was present in about 30% of MCD cases, but absent in TLE. Group analysis localized this anomaly to the medial-most portion of the hippocampus corresponding to CA4-DG. Notably, in a detailed MRI single-case analysis, Montenegro et al. (25) also reported enlarged hippocampi in a few patients with lissencephaly and subcortical band heterotopia. A possible explanation may be that dentate hypertrophy reflects altered gyral morphology with increased number of folds (1, 36). Furthermore, there is evidence from animal models that hypertrophic dentate neurons lead to enlargement of the hippocampus visible to the naked eye (37). Indeed, in a mouse model of cortical dysplasia, mutants with a deletion of the PTEN gene, a tumor suppressor, overactivate the mTOR signaling cascade (37). Normal activity of this pathway is essential for control of cell growth, proliferation, and survival. Its overexpression would induce neuronal hypertrophy and dysmorphism in the hippocampus and the neocortex through various feedback loops. Noteworthy, enhanced mTOR signaling was also identified in hemimegalencephaly (38), and in enlarged dysmorphic neurons and balloon cells of Taylor-type FCD (39) and cortical tubers (40). These hypotheses remain speculative at this moment and future histopathological studies are needed to further clarify the pathophysiology of hippocampal hypertrophy in MCD.

The term malrotation has been coined to describe a set of positioning features characterized by an abnormal rotation of the hippocampus around its long axis (vertical orientation) and a displacement toward the mid-sagittal plane (medial positioning), which were originally evaluated on coronal MRI cuts (1, 3, 7, 41), and later quantified through 3D models (7, 8). Notably, however, a medial shift of the hippocampal centroid may also result from atrophy at the lateral border corresponding to the location of CA1, as shown by our current data. In our earlier analyses, we circumvented this confound by using volume as a nuisance factor (7). Here, position vectors and curvature derived from the mean meridian axis allowed quantifying intrinsic positioning without the need for extra-hippocampal references and revealed that in TLE medial positioning results from bending of the posterior hippocampus ipsilateral to the focus. In MCD, on the other hand, this anomaly was due to supero-medial shift of the left hippocampal body, regardless of the type of the malformation and the lesion side. These findings suggest that mechanisms underlying medial positioning diverge between the two epilepsy syndromes. It is temping to propose that in TLE abnormal positioning may be related to epileptogenic process, whereas in MCD hemisphere-specific vulnerability to injury may be linked to asymmetric brain development (42). In this scenario, abnormalities would occur during a time window of left hemisphere susceptibility when right hemisphere homologs, due to a more advanced stage of development, are relatively spared. Alternatively, the location of the MCD may influence hippocampal morphology through differential connectivity patterns.

Overall, our group-based results suggest that the differential patterns of morphological makeup between TLE and MCD may stem from a complex interplay between genetics, neurogenesis, and epileptogenesis. The methodological framework we propose provides the basis for future individual-based analysis and serial measurements, which may be used as potential clinical and therapeutic diagnostic biomarkers.

## Conflict of Interest Statement

The authors declare that the research was conducted in the absence of any commercial or financial relationships that could be construed as a potential conflict of interest.
